# Global elimination of leprosy by 2020: are we

**DOI:** 10.1186/s13071-015-1143-4

**Published:** 2015-10-22

**Authors:** David J. Blok, Sake J. De Vlas, Jan Hendrik Richardus

**Affiliations:** Department of Public Health, Erasmus MC, University Medical Center, P.O. Box 2040, Rotterdam, CA 3000 The Netherlands

## Abstract

**Background:**

Every year more than 200,000 new leprosy cases are registered globally. This number has been fairly stable over the past 8 years. WHO has set a target to interrupt the transmission of leprosy globally by 2020. The aim of this study is to investigate whether this target, interpreted as global elimination, is feasible given the current control strategy. We focus on the three most important endemic countries, India, Brazil and Indonesia, which together account for more than 80 % of all newly registered leprosy cases.

**Methods:**

We used the existing individual-based model SIMCOLEP to predict future trends of leprosy incidence given the current control strategy in each country. SIMCOLEP simulates the spread of *M. leprae* in a population that is structured in households. Current control consists of passive and active case detection, and multidrug therapy (MDT). Predictions of leprosy incidence were made for each country as well as for one high-endemic region within each country: Chhattisgarh (India), Pará State (Brazil) and Madura (Indonesia). Data for model quantification came from: National Leprosy Elimination Program (India), SINAN database (Brazil), and Netherlands Leprosy Relief (Indonesia).

**Results:**

Our projections of future leprosy incidence all show a downward trend. In 2020, the country-level leprosy incidence has decreased to 6.2, 6.1 and 3.3 per 100,000 in India, Brazil and Indonesia, respectively, meeting the elimination target of less than 10 per 100,000. However, elimination may not be achieved in time for the high-endemic regions. The leprosy incidence in 2020 is predicted to be 16.2, 21.1 and 19.3 per 100,000 in Chhattisgarh, Pará and Madura, respectively, and the target may only be achieved in another 5 to 10 years.

**Conclusions:**

Our predictions show that although country-level elimination is reached by 2020, leprosy is likely to remain a problem in the high-endemic regions (i.e. states, districts and provinces with multimillion populations), which account for most of the cases in a country.

**Electronic supplementary material:**

The online version of this article (doi:10.1186/s13071-015-1143-4) contains supplementary material, which is available to authorized users.

## Background

Leprosy or Hansen disease is caused by an infection of *Mycobacterium leprae,* usually acquired through contact with an infected person. However, not everyone exposed to an infected contact will eventually develop the disease [1]. Worldwide, more than 200,000 new leprosy cases are detected annually [2]. This number has been fairly stable in the past 8 years, indicating ongoing transmission. In 2013, 14 countries reported more than 1000 new cases, of which three countries - India, Brazil and Indonesia - account for more than 80 % of all the cases in the world [2]. The distribution of leprosy is becoming localized to a limited number of countries [2]. Also within countries leprosy is found to be spatially unevenly distributed [3, 4].

The spread of leprosy in a population is highly dependent of the variation in susceptibility of individuals and the intensity of contact with other individuals [5, 6]. As a result leprosy patients are often found to be spatially clustered in regions, neighborhoods, families and households [7–9]. Contacts closest to the index patient, in particular household contacts, have the highest risk [5, 8].

Leprosy patients are treated with a combination of antibiotics known as multidrug therapy (MDT), which effectively heals the patient and thereby also reduces the infectivity in the community. However, MDT alone is not sufficient to prevent new cases. Therefore the main principles of leprosy control also include early detection of leprosy cases [2]. Other methods of prevention such as immunoprophylaxis and chemoprophylaxis against *M. leprae* are not yet widely available [10].

Global elimination of leprosy has been a target for a long time. In 1991, the 44th World Health Assembly already adopted the target of elimination as a public health problem, defined as reducing the prevalence to less than 1 case per 10,000 population by the year 2000 [11]. Although this target was met at a global level, elimination of leprosy as a public health problem has not been achieved in some endemic countries, in particular at a subnational level [2]. Recently, WHO has formulated a roadmap for 17 neglected tropical diseases, including leprosy, to reduce their global impact. The targets for leprosy are (1) global interruption of transmission or elimination by 2020, and (2) reduction of grade-2 disabilities in newly detected cases to below 1 per million population at global level by 2020 [12].

The aim of this study is to investigate whether the first target is feasible given the current control strategy. To be able to assess this, we interpreted the first target of 'interruption of transmission' in terms of incidence reduction, contrary to the original WHO elimination target (as public health problem), which was defined in terms of prevelance reduction. Our operational definition of global elimination of leprosy as applied in this paper is: 'less than 10 new cases per 100,000 population. We focus on the three countries with the highest number of annual new cases: India, Brazil and Indonesia. Since leprosy is spatially unevenly distributed within countries, we will for comparison also make predictions for one high-endemic region within each country: Chhattisgarh (India), Pará State (Brazil) and Madura Island (Indonesia). We will use the existing individual-based model SIMCOLEP, which simulates the transmission and control of leprosy in a population structured by households [6, 13]. This model has been previously quantified to the leprosy situation in northwest Bangladesh with the aim of making future predictions of leprosy trends and testing the impact of various interventions [14].

In this study, we apply SIMCOLEP to make predictions of future leprosy incidence in India, Brazil, Indonesia and aforementioned regions until the year 2030. For each of these countries and regions, we will assess whether elimination, defined as less than 10 new cases per 100,000, will be met by 2020 given continuation of the current control strategies of MDT treatment and early detection.

## Methods

### Model

The individual-based model SIMCOLEP simulates the spread of *M. leprae* in a population structured in households. Dynamics of the population are described by births, movements between households, and deaths. Births are determined by birth rates and newborns are placed in the household of their mother. Movements of individuals to another newly created or existing household occur after marriage, during adolescence or after becoming a widow(er). Deaths are determined by death rates [6].

In the model, transmission of *M. leprae* occurs after direct contact with an infectious individual. Two transmission processes are modelled separately: transmission in the general population and a within-household transmission. Infectivity is determined by the product of the contact rate and the probability of infection during a contact. Each transmission process has its own contact rate: general population, *c*_*pop*_, and within households, *c*_*hh*_. An infected individual will develop either paucibacillary leprosy (PB) or multibacillary leprosy (MB). Both types can be detected, treated and cured, but only MB leprosy is considered infectious in the model. The natural history of leprosy is modelled following the model of Meima *et al.* [15].

Leprosy can only be acquired by susceptible individuals. It is assumed that the majority of the population is never susceptible to leprosy [6]. Since the mechanism that underlies susceptibility is still unknown and could not be identified in previous (modelling) work, susceptibility of an individual is randomly determined at birth. We assumed that 20 % of the population is susceptible (i.e. 80 % is never susceptible), based on model fitting results of a previous study [6]. The type of leprosy (i.e. MB or PB) is randomly determined based on the distribution of the type of leprosy in each country. The MB proportion is about 48 %, 66 %, and 83 % in India, Brazil, and Indonesia, respectively.

### Model fitting and data

The model was fitted to the leprosy situation in India, Brazil and Indonesia, and to one high-endemic region in each of those countries. First, we fitted the population of each country to simulate the overall population and household structure. Population sizes differed between countries and regions: India (1.2 billion), Chhatisgarh (27 million), Brazil (200 million), Pará State (8 million), Indonesia (250 million), Madura (3.6 million) (See Additional file 1: Figure S1). The population in each country and region is quantified using country-specific demographic data as input, including: population growth rates, birth rates, death rates, fertility rates and the age distribution. These data were obtained from various sources including country census, Demographic and Health surveys (DHS) and WHO (see Table 1). Parameters that regulate movements of individuals between households were calibrated such that the simulated distribution of household size matched the observed distribution. Non-married young males can move out of their parent’s household to either start their own household or move to another household. The age of moving is 12 to 22 years in Brazil and Indonesia and 18 to 28 years in India. The fraction of non-married males that moves during adolescence is 98 %, 75 %, and 100 % in India, Brazil and Indonesia respectively. One percent of these moving males in Brazil and none in the other countries will create their own household. The other moving males will go to an existing household, which is randomly determined weighed by size of the households following a Triangular distribution (India: Tri(0,4,3); Brazil and Indonesia: Tri(0,4,2). In all countries all widows and widowers in one person households move to multi person households (i.e. children) at the moment of becoming widow(er). The quantifications of other household parameters were similar to previous work (See Addtional file 1: Table S1) [6]. We assumed that household structures in each region were similar to the household structures of the whole country. Data on household size distributions were obtained from DHS. Goodness of fit of the distribution of household size was evaluated by a Chi-square test.

**Table 1:** Overview of data used to quantify the model

	India		Brazil		Indonesia	
	Years	Source	Years	Source	Years	Source
Demographic data:						
*Population growth*	1901–2011	Census India	1872–2010	Census Brazil	1850–2010	Census Indonesia (BPS)
*Fraction married*	2001, 2011	Census India	1990, 2000, 2010	IBGE^c^	1993, 1997, 2000, 2007	IFLS^e^
*Survival rates*	1990, 2000, 2012	WHO	1991, 2000, 2010	IBGE^c^	1990, 2000, 2009	WHO
*Fertility rates*	1992, 1998 , 2006, 2011, 2012, 2013	DHS^a^, Census India	1991, 1995, 2000, 2005, 2010	IBGE^c^	1971, 1980, 1990, 1991, 1997, 2003, 2007, 2012	DHS^a^
*Age distribution*	2013	DHS^a^	1990, 2000, 2010	IBGE^c^	2012	DHS^a^
*Distribution of household size*	1993, 1999, 2006	DHS^a^	1990, 2000	IBGE^c^	1991, 1994, 1997, 2003, 2007	DHS^a^
Epidemiological data:						
*New case detection rate*						
*- Country*	1991–2015	NLEP^b^	1990–2014	SINAN^d^	2000–2013	NLR^f^
*- High-endemic region*	2008–2015	NLEP^b^	1990–2014	SINAN^d^	2001–2010	NLR^f^
*MB proportion*	2011–2013	NLEP^b^	2001–2012	SINAN^d^	2000–2013	NLR^f^
*BCG coverage*	1980–2013	WHO	1980–2013	WHO	1980–2013	WHO

^a^Demography and Household Survey; ^b^National Leprosy Elimination Programme; ^c^Brazilian Institute of Geography and Statistics; ^d^Sistema de Informações de Agravos de Notificação; ^e^Indonesian Family Life Survey; ^f^Netherlands Leprosy Relief foundation

After fitting the population and household structure in each country, we fitted the simulated leprosy trends to the observed trends in each country and region separately. Data used to fit leprosy trends include the new case detection rate and the MB proportion. Indian data were obtained from the *National Elimination Program* (NLEP) [16]. Brazil leprosy data came from the *Sistema de Informações de Agravos de Notificação* (SINAN) database [17]. The SINAN database is the Brazilian national database for communicable diseases. Finally, leprosy data for Indonesia were provided by the *Netherlands Leprosy Relief* (NLR) foundation (see Table 1).

In order to fit the leprosy trends, we also modeled leprosy control programs of the past decades in each country. The leprosy control program consisted of treatment with Dapsone until 1989 and MDT afterwards, and passive and active case detection. Changes in passive detection were expressed in terms of detection delays. These detection delays were fitted to match the trends of the new case detection rates. Based on literature, most recent detection delays were set to 2–3 years in our model [18]. The estimated detection delays gradually improved since 1970 from 13 to 2 years, 18 to 3 years, and 10 to 2 years (gamma distributed) in India, Brazil and Indonesia, respectively. Active case detection, defined as examining contacts of the patient, was only included if data about coverage rates were available. In Indonesia active detection was included since 2010 with a coverage rate of 9–11 %. In Brazil the coverage rate was between 43 and 59 %, and started since 2003 [19]. No active case detection was included in India [20]. BCG vaccination in infants, which has a protective effect of 60 %, was also included [21]. Coverage rates of BCG vaccination in infants, obtained from WHO, were used [22]. We further assumed that the national leprosy control strategy was implemented consequently within regions.

Leprosy new case detection rates were fitted to the data by calibrating contact rates. In this study, we only calibrated the contact rate in the general population. Since no data was available about the prevalence and/or incidence of leprosy by household size and per household member, we fixed the contact rate within household to the optimal value of previous work (*c*_*hh*_ = 0.98) [6]. We assumed that contact rates within households would not differ between countries and regions. The contact rate in general population (*c*_*pop*_) of each country and region was estimated separately by fitting the simulated new case detection rates to the observed new case detection rates from the data (see Table 1). To this end, the simulated new case detection rates (mean of 100 runs) were compared to the data by a log-likelihood function assuming a Poisson distribution. These likelihood ratios were fitted to a polynomial regression meta-model to obtain the optimal *c*_*pop*_ value.

New simulations were performed with the optimal *c*_*pop*_ values. A detailed description of the fitting procedure can be found elsewhere [6]. A manual of the model, the model itself and the input files with country-specific quantifications are provided in Additional file 2 and Additional file 3.

### Future projections

Using the best fit for each country and high-endemic region, we made future predictions assuming continuation of current leprosy control programs: MDT treatment, passive detection and active detection (only Brazil and Indonesia). Each simulation run starts in the year 1000 with a modelled population size of 20,000 individuals that remains constant until 1850–1900. Afterwards the population increase with the annual growth rate to more than 125,000 individuals in 2030. All simulations continued to the year 2030 to predict future trends and to determine whether elimination could be reached by 2020. Simulation results were an average of 100 runs.

## Results

**Fig. 1:**
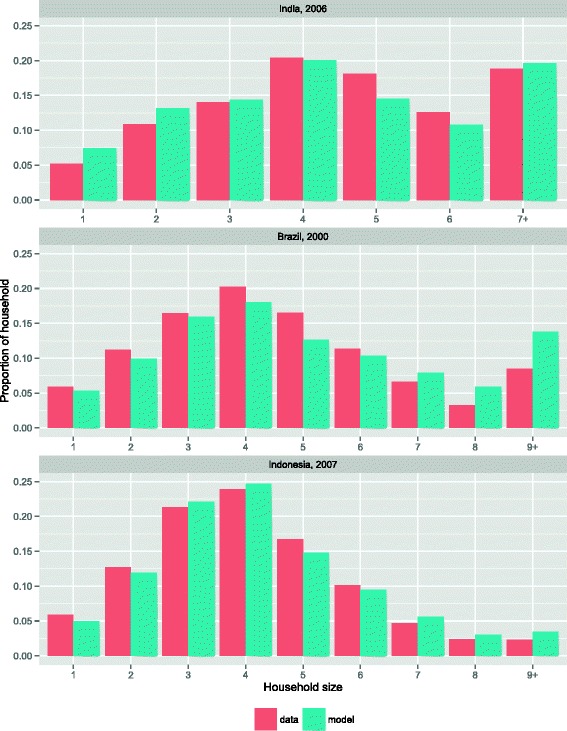
Result of calibration of the household structure in the population to the observed distribution of household size in India (2006), Brazil (2000), and Indonesia (2007). There is no significant difference between data and the simulated distributions (χ^2^-test)

**Fig. 2:**
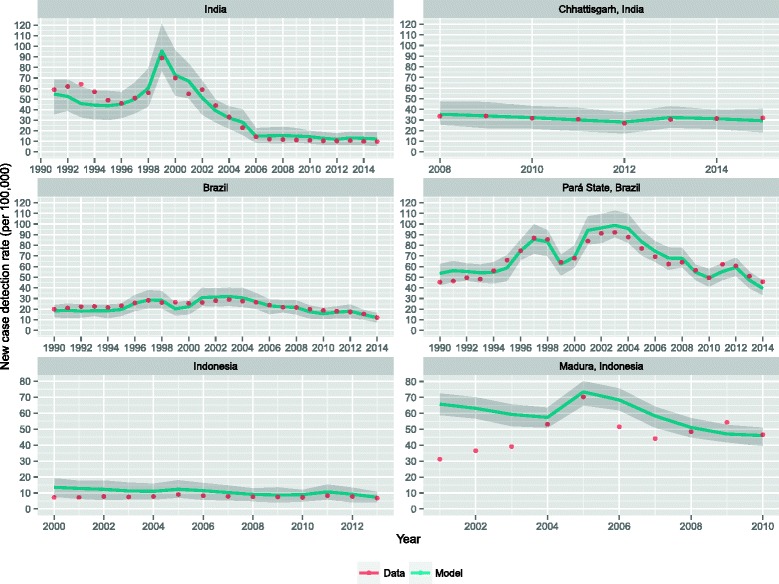
Result of fitting the new case detection rates to the observed new case detection rate. The solid lines are the result of an average of 100 runs. The shaded area is the 90 % confidence interval (stochastic variation in individual runs)

Results of calibration of the household structure in the population are shown in Figure 1. In each country, the simulated household structure closely resembled the observed distribution of household sizes. Using these household structures, the trends in leprosy new case detection rates were fitted to the observed data. The optimal values of the contact rates are: 0.970 (95 % CI: 0.929-1.011; India), 1.679 (95 % CI: 1.638-1.720; Chhattisgarh), 0.367 (95 % CI: 0.326-0.408; Brazil), 0.543 (95 % CI: 0.503-0.584; Pará State), 0.104 (95 % CI: 0.063-0.145; Indonesia), and 0.235 (95 % CI: 0.194-0.276; Madura). Figure 2 shows that in all scenarios our model was able to reproduce the observed trends. Madura shows a poor fit for the years 2001–2003, given the operational changes in leprosy control that were considered at national level. Also the simulated MB proportion in each country matched the proportion in data (See Additional file 1: Figure S2).

**Fig. 3:**
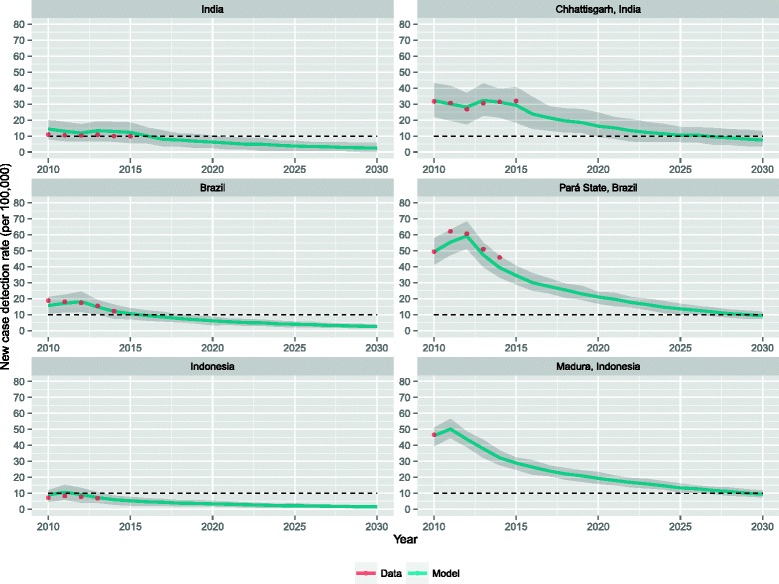
Predicted new case detection rates until 2030 assuming continuation of the present leprosy control strategy. The solid lines are the result of an average of 100 runs. The shaded area is the 90 % confidence interval (stochastic variation in individual runs)

Figure 3 shows the predicted new case detection rate trends until 2030 for India, Brazil and Indonesia, assuming that current national leprosy control programs will continue unchanged. For all three countries a substantial decline in new case detection rate can be observed until 2030. On a country-level, the elimination target of less than 10 per 100,000 has already been met in India and Indonesia, and will be met by 2016 in Brazil. In 2020, the predicted new case detection rates are 6.2 (90 % CI: 2.6-10.2), 6.1 (90 % CI: 3.4-8.7), and 3.3 (90 % CI: 1.8-5.2) new cases per 100,000 in India, Brazil and Indonesia, respectively. The predicted annual decrease between 2015 and 2030 is approximately 10.5 %, 9.1 %, and 8.1 % in India, Brazil and Indonesia, respectively.

The new case detection rates in the regions Chhattisgarh, Pará State and Madura are 2–7 times higher than the national rates. Similarly to country-level predictions, the new case detection rates in the high-endemic regions will continue to drop over time. However, in these regions the elimination target will not be met in time. In 2020, the predicted new case detection rates are 16.2 (90 % CI: 9.4-24.7), 21.1 (90 % CI: 16.9-24.2), and 19.3 (90 % CI: 15.9-23.3) per 100,000 in Chhattisgarh, Pará State, and Madura, respectively. Elimination in these regions will not be established before 2026. The annual decrease between 2015 and 2030 is about 8.7 %, 8.4 %, and 7.2 % in Chhattisgarh, Pará State, and Madura, respectively. Predictions in terms of annual new cases are shown in Additional file 1: Figure S3.

The distribution of new leprosy cases by age group differs by country and is affected by control strategies. Improved detection will mainly increase the detection of older individuals (See Additional file 1: Figure S4). Also the distribution of new leprosy cases by household size differs by country. In Brazil most cases are found in bigger households, while in Indonesia most cases are found in smaller households (See Additional file 1: Figure S5).

## Discussion

We used the individual-based model SIMCOLEP to assess whether the WHO target of elimination of leprosy, defined as less than 10 new cases per 100,000 annually, would be met by 2020. We focused on three high-endemic countries, India, Brazil and Indonesia, which account for more than 80 % of all cases worldwide. Our study shows that elimination of leprosy in these countries has already been met or will be met in the very near future at country-level. However, in the high-endemic regions of these countries – Chhattisgarh, Pará State and Madura – elimination of leprosy will not be achieved by 2020 with the current leprosy control strategy. Regional new case detection rates are 2–7 times higher than at country-level.

Country-level predictions of the new case detection rates, which suggest elimination, provide a biased view of the leprosy situation, because these rates are masked by the large population size of each country. Focusing on high-endemic regions where this elimination target has not yet been achieved will give a more realistic representation of the actual leprosy situation in a country. It also reflects more accurately that the distribution of leprosy is becoming more localized [3, 4, 23].

Our results show a continuous decline in new case detection rates, demonstrating that the present leprosy control strategy, if consistently applied, will reduce transmission. Results also show that confidence bounds become smaller around future projections. This is caused by the downward trend of the NCDR given the control strategy and partially by the population growth. Predictions in terms of annual number of new cases illustrate that confidence bounds decrease less rapidly when the population growth is not considered (See Additional file 1: Figure S3 ). The annual rate of decline is greatest in India, followed by Brazil and Indonesia. Also within countries the speed of decline differs. This is caused by differences in intensity of contact, reflecting different living conditions based on varying socioeconomic and cultural circumstances. In high-endemic regions, which are generally less developed, the calibrated contact rate in the general population was higher than at country-level country rates, which reflects a nation-wide average.

The poorer fit of Madura in Indonesia might be explained by regions specific operational changes in control. Madura is an island in Indonesia and therefore relatively isolated. It has always been a high-endemic region. The poor fitting is probably the result of the focused intensified case finding activities in that particular area between 2003 and 2005 causing the NCDR to double in that region, which our model did not account for [24, 25].

The large number of undetected cases remains a threat to the elimination of leprosy generally. Leprosy is a slow disease with a long incubation time and long delays in detection [20, 26, 27]. These missing cases still contribute to the ongoing transmission. It has been estimated that over 4 million cases are missed between 2000 and 2020 worldwide [27]. It has been argued that in India half of the cases have not been reported to meet the elimination targets of 2005 [20]. Since our model heavily depends on the available case data, including their imperfections, the problem of missing cases is also inherent to our predictions. On the whole, the actual number of new leprosy cases is likely to be higher than presented in our predictions [20, 27, 28].

A concern of this study is that the predictions are made under the assumption that being susceptibility to leprosy was fully randomly determined. Previous work has shown that although this may be a valid assumption, other mechanisms, such as genetic inheritance of susceptibility, might also explain variation in susceptibility [6]. Assuming another mechanism for susceptibility has proven to somewhat slow down the speed of decline of the new case detection trends [14]. Our results can therefore be regarded as a best case scenario.

In our study elimination was defined as less than 10 new cases per 100,000, while elimination is officially defined as a prevalence rate of less than 10 per 100,000 population. This will, however, not alter our conclusions, because the prevalence rate of leprosy does not differ much from the new case detection rate.

Furthermore, our predictions are based on a continuation of the present leprosy control strategy, which also includes some contact tracing in Brazil and Indonesia. It assumes full adherence to the strategy. Any unexpected adverse events, such as famines and social upheaval, have not been considered. On the other hand, our study also does not account for possible additional leprosy control practices in regions, such as chemoprophylaxis for contacts of leprosy cases.

An important next step is to determine which interventions at population level would bring elimination forward and have the highest impact on future incidence of disease through the interruption of transmission. The focus should be on interventions targeting the contacts of leprosy patients to prevent new cases [9]. Examples of such intervention are intensive contact tracing, administering chemoprophylaxis or immunoprophylaxis (e.g. BCG-like leprosy vaccine) and early diagnosis of leprosy using diagnostic tests for infection or tests that predict clinical disease.

## Conclusion

Although it seems that country-level elimination is reached by 2020, leprosy still remains a problem in the high-endemic regions, which account for most of the cases in a country. These regions often have a multimillion population in which rapid progress of leprosy control, even if conducted optimally, will not be achieved soon. We therefore conclude that ongoing transmission of *M. leprae* will make global elimination of leprosy as a unlikely to occur by 2020 without further control measures.

## Additional files

Additional file 1: Table S1. – Overview of parameters to model household movements. **Table S2.** – Overview of parameters to model leprosy. **Figure S1.** – Population size from 1820 to 2030. **Figure S2.** – Simulated new case detection rate trends by leprosy classification (PB and MB). **Figure S3.**– Predicted annual number of new cases until 2030 assuming continuation of the present leprosy control strategy. **Figure S4.**– Simulated age distribution of new leprosy cases over time. **Figure S5.** – Simulated household size distribution of new leprosy cases over time. (DOCX 123 kb) Additional file 2:
**SIMCOLEP manual.** SIMCOLEP manual gives a complete overview of all parameters and functions in the model. It also provides a description of the input file. (DOCX 59 kb) Additional file 3:
**SIMCOLEP model with input files.** Zip archive contains: Instruction file, SIMCOLEP model (version 1.4.12), Input files for India, Brazil and Indonesia, Source code, R-script for output. The contents of this zip archive are licensed under the Creative Commons Attribution-NonCommercial-NoDerivatives 4.0 International License. To view a copy of this license, visit http://creativecommons.org/licenses/by-nc-nd/4.0/ or send a letter to Creative Commons, PO Box 1866, Mountain View, CA 94042, USA. By opening Additional file 3, you agree to the aforementioned license. You are free to use and share (copy and redistribute the material in any medium or format) the material contained within Additional File 3 under the following terms: Attribution — You must give appropriate credit, provide a link to the license, and indicate if changes were made. You may do so in any reasonable manner, but not in any way that suggests the licensor endorses you or your use. NonCommercial — You may not use the material for commercial purposes. NoDerivatives — If you remix, transform, or build upon the material, you may not distribute the modified material. 
