# Supplementary material for: Global elimination of leprosy by 2020: are we
Source: Parasit Vectors. 2015 Oct 22;8:548. doi: 10.1186/s13071-015-1143-4 (PMC4618543; doi:10.1186/s13071-015-1143-4)
Supplement: Additional file 1: Table S1. — – Overview of parameters to model household movements. Table S2. – Overview of parameters to model leprosy. Figure S1. – Population size from 1820 to 2030. Figure S2. – Simulated new case detection rate trends by leprosy classification (PB and MB). Figure S3.– Predicted annual number of new cases until 2030 assuming continuation of the present leprosy control strategy. Figure S4.– Simulated age distribution of new leprosy cases over time. Figure S5. – Simulated household size distribution of new leprosy cases over time. (DOCX 123 kb) [file 13071_2015_1143_MOESM1_ESM.pdf]

Additional file 1: Supplementary tables and figures

David J. Blok[1], Sake J. de Vlas[1], Jan Hendrik Richardus[1]

[1]Department of Public Health, Erasmus MC, University Medical Center Rotterdam, The Netherlands

Contents

Table S1 – Overview of parameters to model household movements 2
Table S2 – Overview of parameters to model leprosy 3
Figure S1 – Population size from 1820 to 2030. 4
Figure S2 – Simulated new case detection rate trends by leprosy classification (PB and MB). 5
Figure S3 – Predicted annual number of new cases until 2030 assuming continuation of the present leprosy control strategy........ 6
Figure S4 – Simulated age distribution of new leprosy cases over time. 7
Figure S5 – Simulated household size distribution of new leprosy cases over time. 8
References 9

Table S1 – Overview of parameters to model household movements

Parameters	Values	Source
**Household movements**		
Fraction of non-married individuals moving randomly	India and Chattisgarh: *0.98 (only males)*<br>Brazil and Para State: *0.75 (only males)*<br>Indonesia and Madura: *1.0 (only males)*	Calibrated
Age range of random movements of non-married individuals	India and Chattisgarh: *18-28yrs*<br>Brazil and Para State: *12-22yrs*<br>Indonesia and Madura: *12-22yrs*	Calibrated
Household size to move to	India and Chattisgarh: *Triangular(0,4,3)*<br>Brazil and Para State: *Triangular(0,4,2)*<br>Indonesia and Madura: *Triangular(0,4,2)*	Calibrated
Rate at which households are split after adding a married couple	All: *Exponential(12)*	Fischer et al. 2010
Fraction of individuals moving to child after becoming a widow/widower	All: *1.0*	Calibrated
Fraction moving to partner after marriage	All: Males: *0.0*; Females: *0.75*	Fischer et al. 2010
Fraction of moving person that create their own household	India and Chattisgarh: *0.0*<br>Brazil and Para State: *0.01*<br>Indonesia and Madura: *0.0*	Calibrated

Table S2 – Overview of parameters to model leprosy

Parameters	Values	Source
**Natural History of infection**		
Susceptible	20%; Allocation randomly determined	Fischer et al. 2010
MB/PB proportion	India and Chattisgarh: *PB 52%; MB 48%*<br>Brazil and Para State: *PB 34%; MB 66%*<br>Indonesia and Madura: *PB17%; MB 83%*	NLEP[a]<br>SINAN[b]<br>NLR[c]
PB asymptomatic state	Mean duration: *4.2 yrs (SD: 1.9); Gamma distributed*	Fine, 1982; Meima et al. 2004
PB recovered	Mean duration: *5 yrs; Exponentially distributed*	Fischer et al. 2010
MB asymptomatic	Mean duration: *11.1 yrs (SD: 5.0); Gamma distributed*	Fischer et al. 2010
**Treatment**		
Treatment	Dapsone: 1970-1990<br>Dapsone relapse: 0.015 y^{-1}<br>MDT: since 1990<br>MDT relapse: $0.001y^{-1}$<br><br>Relapse to MB: 90%<br>Relapse to PB: 10%	International Leprosy Association Technical Forum, 2002
**Control**		
Active case detection	India and Chattisgarh: *none*<br>Brazil and Para State: *since 2003; coverage 0.59*<br>Indonesia and Madura: *since 2009; coverage 0.12*	Lockwood et al. 2014<br>Ministry of Health, Brazil<br>NLR[c]
Passive case detection	India and Chattisgarh: *1970: 13yrs; 1980: 11yrs; 1990: 9yrs; 1997: 8.5yrs; 1998: 5.5yrs; 1999: 3yrs; 2001: 2yrs; 2005: 3yrs; 2012: 2.5yrs; After 2014: 2yrs*<br>Brazil and Para State: *1970: 18yrs; 1990: 16yrs; 1996: 15yrs; 1997: 14yrs; 1998: 12yrs; 2001: 10.5yrs; 2002: 9yrs; 2003: 5.5yrs; 2008: 4.5yrs; After 2011: 3yrs*<br>Indonesia and Madura: *1970: 10yrs; 1980: 8yrs; 1989: 5.5yrs; 1997: 5yrs; 1998:5.5yrs; 2004: 4.5yrs; After 2011: 2yrs*	Calibrated
BCG coverage in infants	Protective effect of 60%	Schuring et al. 2009
**Transmission**		
Contact rate in the general population	India: *0.970; 95% CI: 0.929-1.011*<br>Chhattisgarh: *1.679; 95% CI: 1.638-1.720*<br>Brazil: *0.367; 95% CI: 0.326-0.408*<br>Pará State: *0.543; 95% CI: 0.503-0.584*<br>Indonesia: *0.104; 95% CI: 0.063-0.145*<br>Madura: *0.235; 95% CI: 0.194-0.276*	Calibrated
Contact rate within households	0.98	Fischer et al. 2010
Infectivity	PB: *not infectious*<br>MB: *Asymptomtic: 0-1 (linear increase); Symptomatic: 1 (constant)*	Meima et al. 2004

[a]National Leprosy Elimination Programme; [b]Sistema de Informações de Agravos de Notificação; [c]Netherlands Leprosy Relief foundation

Figure S1 – Population size from 1820 to 2030.
The solid line is the exponential growth curve used as input for the model.

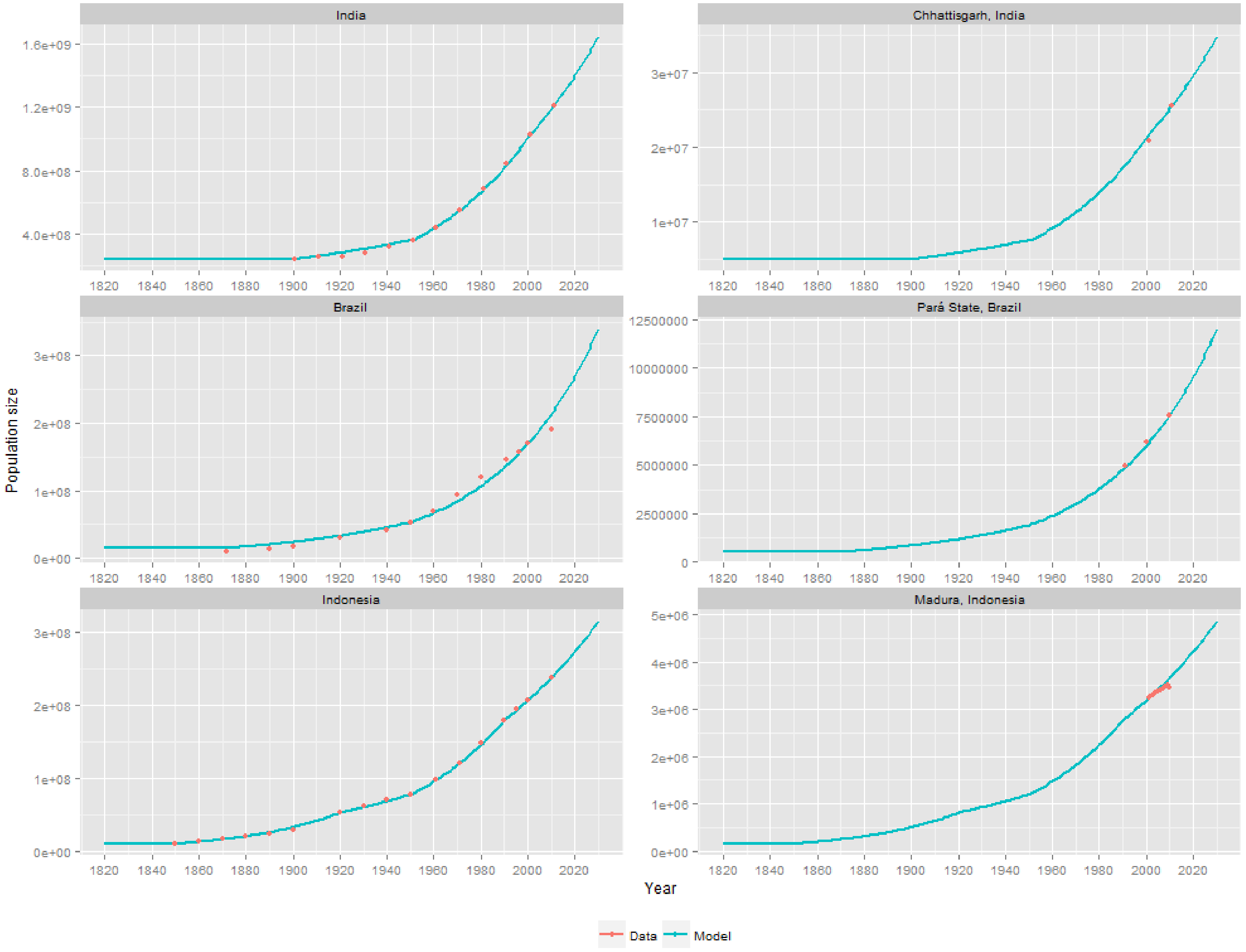

Figure S2 – Simulated new case detection rate trends by leprosy classification (PB and MB). Simulated MB proportions matched the MB proportions from the data (India: 48%, Brazil: 66%, Indonesia: 83%)

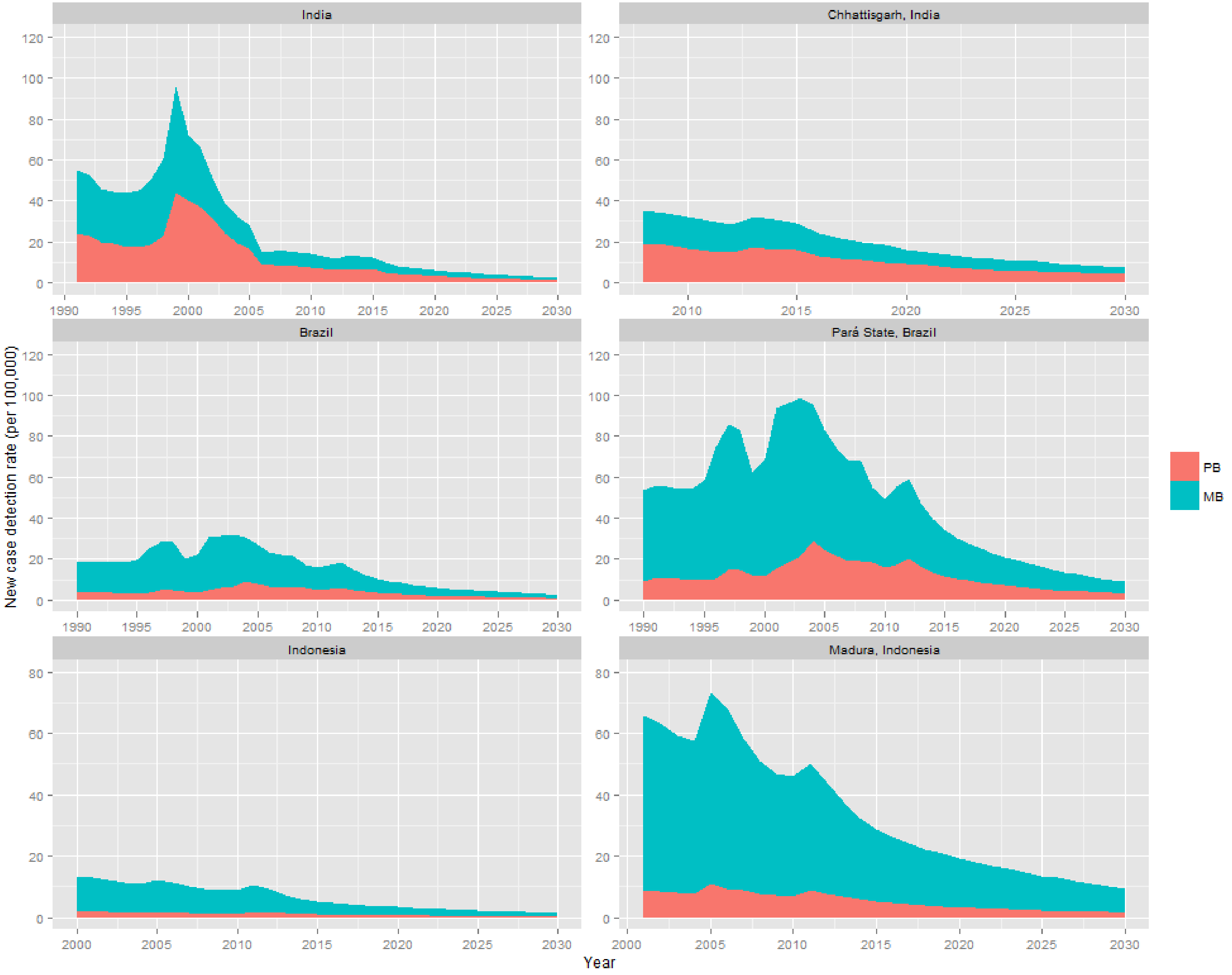

Figure S3 – Predicted annual number of new cases until 2030 assuming continuation of the present leprosy control strategy.

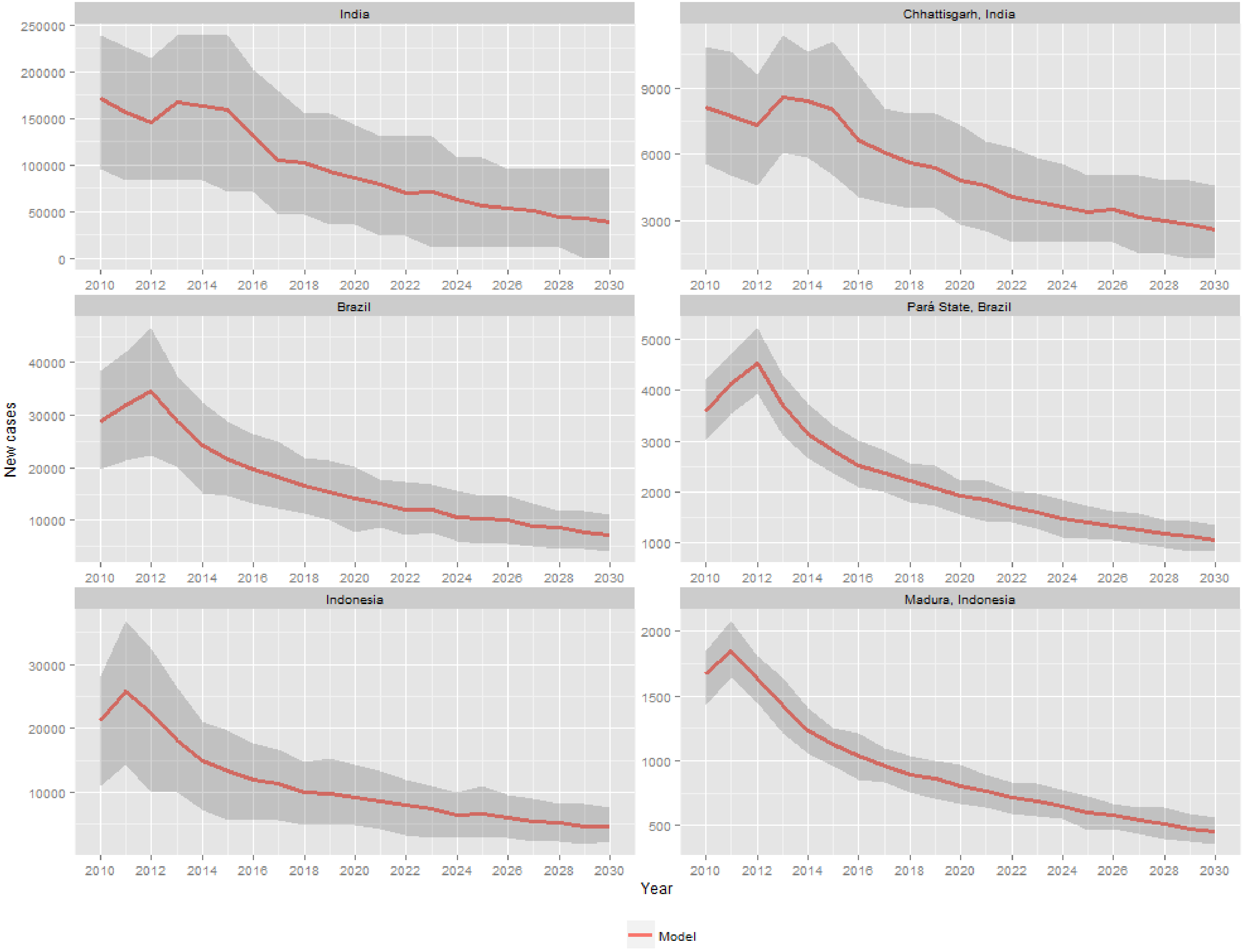

Figure S4 – Simulated age distribution of new leprosy cases over time.

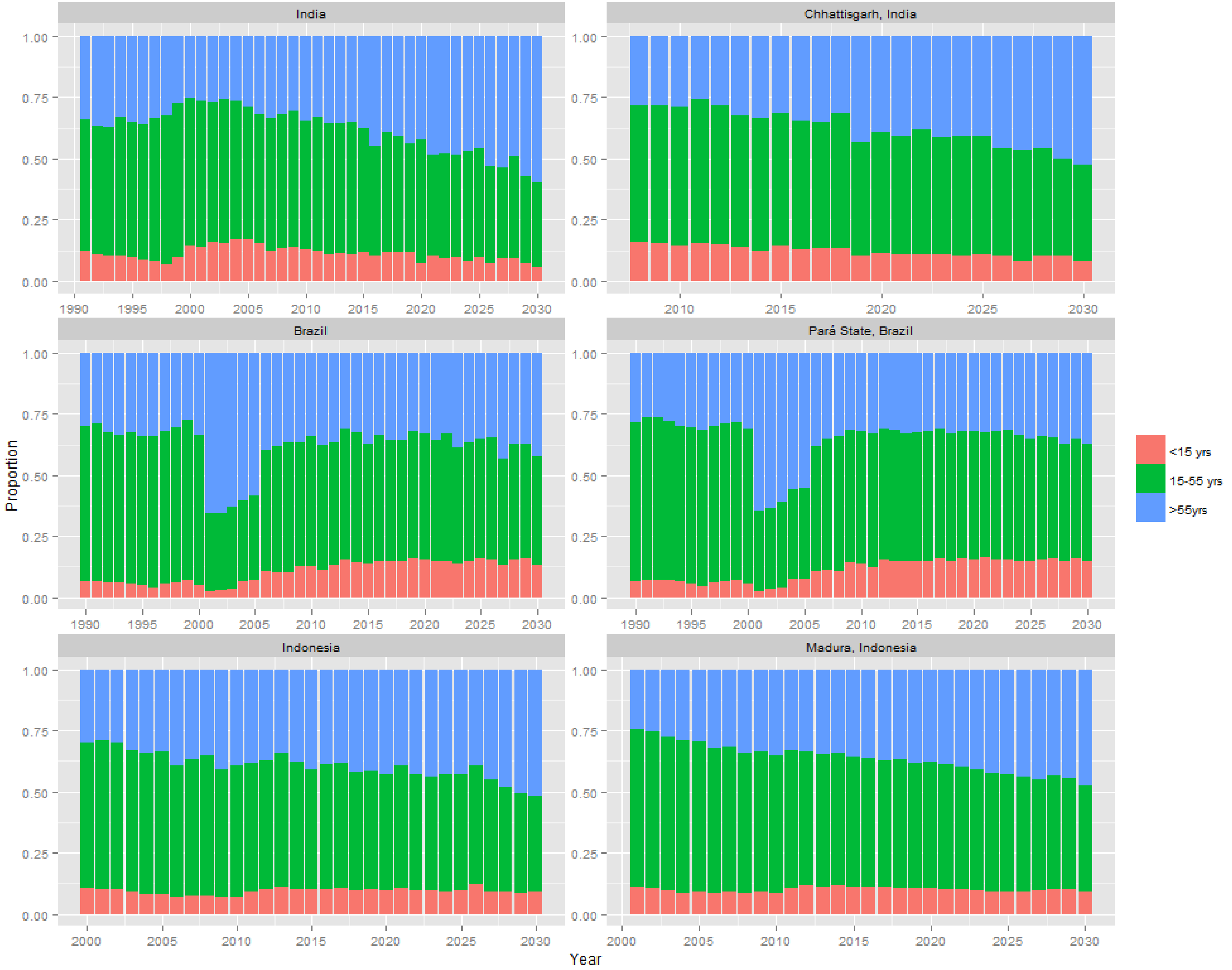

Figure S5 – Simulated household size distribution of new leprosy cases over time.

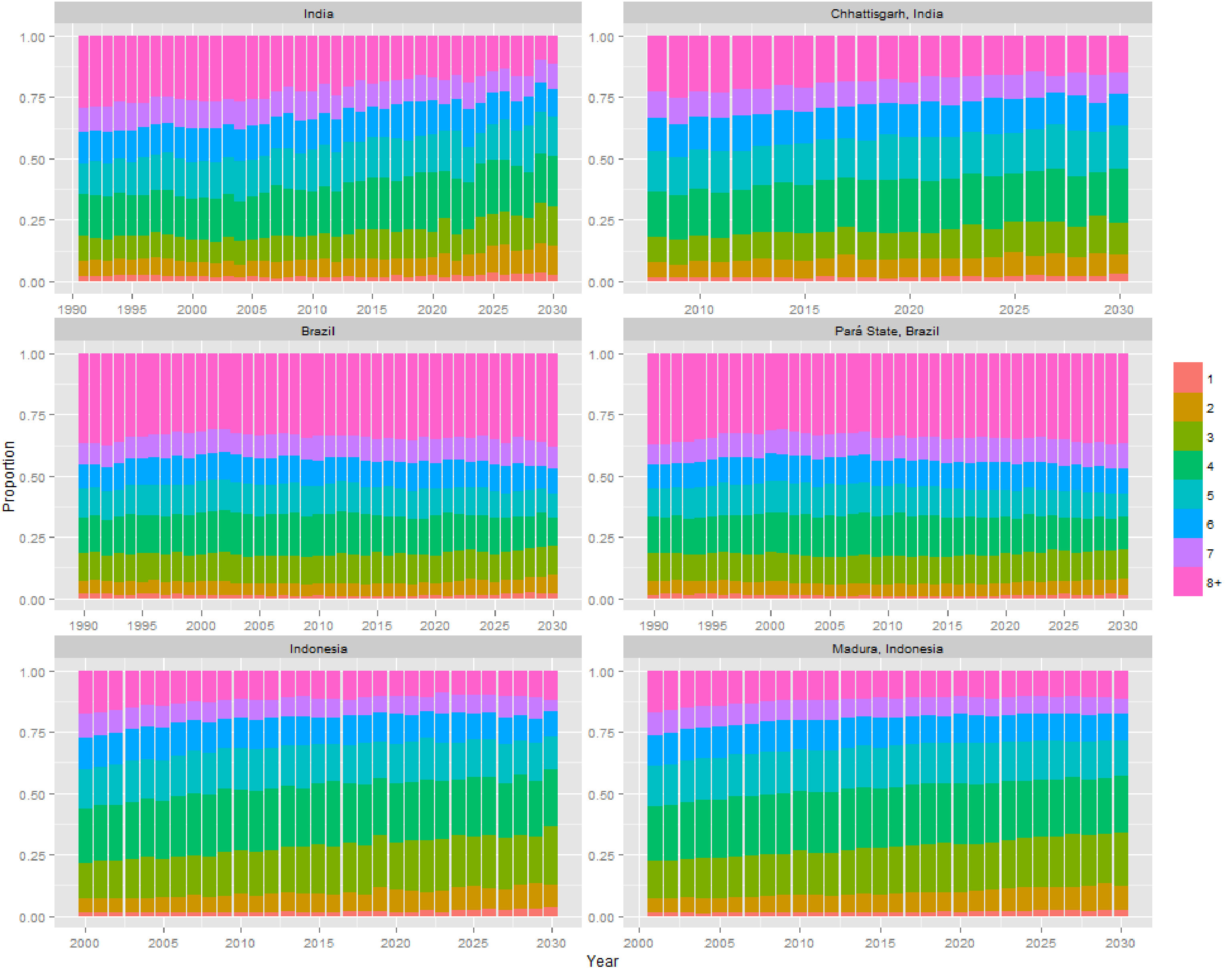

References

1. Fischer, E.A.J., et al., Different mechanisms for heterogeneity in leprosy susceptibility can explain disease clustering within households. PLoS One, 2010. 5(11): p. e14061.

2. Fine, P.E., Leprosy: the epidemiology of a slow bacterium. Epidemiological Review, 1982. 4: p. 161-188.

3. Meima, A., et al., The future incidence of leprosy: a scenario analysis. Bull World Health Organ, 2004. 82(5): p. 373-80.

4. Anonymous, Report of the International Leprosy Association Technical Forum. Internationl Journal of Leprosy and other Mycobacterial diseases, 2002. 70: p. S1-S62.

5. NLEP. National leprosy elimination programme. Available from: http://nlep.nic.in/.

6. SINAN. Sistema de Informações de Agravos de Notificação. Available from: http://portalsaude.saude.gov.br/.

7. Lockwood, D.N., V. Shetty, and G.O. Penna, Hazards of setting targets to eliminate disease: lessons from the leprosy elimination campaign. BMJ, 2014. 348: p. g1136.

8. Ministry of Health, National Hansen's Disease Control Programme Report 2009-2010. 2011, Ministry of Health.

9. Schuring, R.P., et al., Protective effect of the combination BCG vaccination and rifampicin prophylaxis in leprosy prevention. Vaccine, 2009. 27(50): p. 7125-8.
