# Supplementary material for: Global elimination of leprosy by 2020: are we
Source: Parasit Vectors. 2015 Oct 22;8:548. doi: 10.1186/s13071-015-1143-4 (PMC4618543; doi:10.1186/s13071-015-1143-4)
Supplement: Additional file 2: — SIMCOLEP manual. SIMCOLEP manual gives a complete overview of all parameters and functions in the model. It also provides a description of the input file. (DOCX 59 kb) [file 13071_2015_1143_MOESM2_ESM.pdf]

Additional file 2: Manual of SIMCOLEP version 1.4.12

Author: Egil A.J. Fischer

Table of contents

Introduction 3

XML-tags 3

Functions and Distributions 3

Functions that can be used as distribution 3

Combined functions and distributions 3

Output codes 4

Input for the model 5

Simulation 5

Transmission 5

Population 5

Movement 6

Natural history of infection 6

Susceptibility 7

Introduction of infection 9

Interventions 9

Treatment 10

Active Detection 10

TABLES' for DEMOGRAPHY 12

NEIGHBOURHOOD 12

INITIAL DEMOGRAPHIC DISTRIBUTIONS 12

Seeds 12

Settings 13

Introduction

The SIM*CO*LEP input file is an xml-file. It is subdivided into several sections. Each of these sections requires a number of obligatory functions, distributions parameters or settings. Although it is not compulsory to divide the input file into these sections it makes it more readable and here it will be discussed in this divisions.

XML-tags

The SIM*CO*LEP xml input file subdivided into groups of functions, parameters, methods and seeds using the following tags:
<Functions>: In these sections functions and distributions are defined.
<Parameters>: In these sections parameters are defined
<Methods>: In these sections methods are defined
<Seeds> : In this section the seeds are defined for the random number generator.
<Files>, <Path> and <Output>: These sections define the path, file an type of output generated
Other tags are the name of the input parameter or function or define the type of function or distribution (see section Functions and Distributions).

Functions and Distributions

Functions and distributions can have different types. Here follows a list of functions/distributions using a typical setting:

Functions that can be used as distribution

Constant: <f1 type="Constant" c="400" />
Exponential: <f2 type="Exponential" N0="400" r="0.007" t0="800" />
Uniform: <f type="Uniform" min="12" max="22" Yvalue="0.3/10" />
Triangular: <f type="Triangular" start="0.0" end="50.0" max="4.0" />
Erlang: <f type="Erlang" m="5" r="5/4.17854" />

QFraction: <f type="QFraction" cum="false" interpolate="false">
<e min="0" value="0.21875/0.24375" />
<e min="1" value="0.02/0.24375" />
<e min="2" value="0.005/0.24375" />
</f> *NB QFraction should always add up to one.*

QDiscreteFunction: <f type="QDiscreteFunction" interpolate="false" cum="false">
<entry min="0" value="0.000" />
<entry min="1" value="0.500" />
<entry min="2" value="0.750" />
</f>

Combined functions and distributions

These combine several function/distributions for different time intervals. If necessary interpolation over time intervals is performed.

cFunction: <f type="cFunction">
<interval_starts t1="0" t2="800" t3="950" />
<f1 type="Constant" c="400" />
<f2 type="Exponential" N0="400" r="0.007" t0="800" />
<f3 type="Exponential" N0="400x2.858" r="0.0232" t0="950" />

</f>

NB: Not a distribution. Note that you can use the multiplication sign “x” in parameter values of functions.

TimeDistribution: <f type="TimeDistribution">
<interval_starts t1="0" t2="970" t3="975” />
<d1 type="Constant" c="110" />
<d2 type="Erlang" m="2" r="2/12" />
<d3 type="Erlang" m="2" r="2/11" />
</f>

Output codes

Output codes are constructs starting with a main indicatory character and then followed by specifications.

Main indicatory characters:

- L: Leprosy statistics based on state of individuals.
- D: Demographic statistics (split up by specifications)
- C: Clinic statistics, which differ from leprosy statics that this only data from the clinic and thus from patients that are ‘seen’ in a clinic
- Q: Statistics per household
- A: Statistics per age-class

Specifications:

- GB: Database will result in an csv-file (requires an output data base for the output)
- p: prevalence
- i: incidence
- s: Sum over runs
- a: Average over runs
- g: Demographics such as age (only with D)
- h: Household statistics (only with D)
- m: Movement statistics (only A and D)

Examples:
<LGB /> places all leprosy statistics in a database. Specify in output-files: <databaseL....\>
<Qs /> Summarizes (leprosy) statistics per household and sums over runs
<Lpa /> Average prevalence of leprosy
<Lis /> Sum of incidence (per time interval) of leprosy
<Dgs /> Sum of demographics

Input for the model

The inputs as described below are required to run the model properly, unless is stated otherwise.

Simulation

Parameters

<RunTime value="1003.0" />	Length of simulation in years
<repeats value="100" />:	Number of times the simulation is repeated
<layers value="50" />:	Number of disease histories used per demographic history *(see paper on MUSIDH)*
<recordStart value="970.0" />	Start recording output at this time
<recordInterval value="1.0" />	Interval between records
<databaseStart value="1000" />	Start adding individuals to the database from this time

Files

<Files>:	Files for output
<out>	Output files
<output value="commondom.txt" />	Cumulative output
<databaseL value="commondom5p.csv" />	Database
</out>	
</Files>	
<Path>	Path for output
<out>	Output path
<output absPath="false" />	absPath=”false” = current directory
</out>	
</Path>	
<!-- Output codes -->	
<Output>	Codes to define which output to produce

Transmission

Parameters

<random_mixing_code value="999" />	The parameter determines which people are at risk for the Poisson process transmission.<br>999: everybody at risk of population infection process<br>777: exclude the own household
<c_pop value=" 7.5 " />	Rate of Poisson process transmission
<c_dwel value=" 0 " />	Infection rate within household

Population

Functions

<popsize type="…">	Size of the population at time t

Parameters

<sexratio value="0.5" />	Well, let’s say that this will often be 0.5.

Movement

Functions

<move_male type="…"/>	Distribution determining the age of moving without marriage for males
<!-- duration to random movement for males-->	
<move_female type=".." />	Distribution determining the age of moving without marriage for females
<levelweight type=".."/>	Weighting function to determine size of the household to which an individual moves
<split type="…" />	Rate at which households are split after adding a married couple

Parameters

<fractMoveWidow value="…" />	Fraction of females moving to child after becoming widow
<fractMoveWidower value="…" />	Fraction of males moving to a child after becoming widower
<movetomale value=".." />	Fraction moving to the male after marriage
<movetofemale value=".." />	Fraction moving to the female after marriage
<movesingle value=".." />	Fraction of random moving person that create their own household
<densityMoveWidow value=".." />	Household size above which widows do not move

Methods

<RMafterM value="on" />:	Movement after marriage allowed. This input is not required and will default to "on"

Natural history of infection

Functions

<DiseaseHistory... type ="*type*"/> : The (...) should be replaced for an integer. If there are for example 4 types (including immune individuals) than specify DiseaseHistory0, Diseasehistory1, .., Diseasehistory3. The "*type*" specifies the type of leprosy.

The type is either: Immune, shPB (self-healing PB), dgPB (degrading PB), or MB. Type dgPB is not well tested. Each type requires some functions:

- Immune: No further functions
- shPB
  - <lat type="Erlang" m="5" r="5/4.17854" />: Length latency (non-infectious) period
  - <incpb type="Constant" c="0.0" />: Length incubation period until PB-disease
  - <sh type="Exponential" r="-1/5" />: Time until self-healing after incubation period
  - <det type="TimeDistribution" ....>: Time until detection after incubation period
  - <kernel type="PBKernel" beta="0.0" />: Infectivity function (incorrectly called kernel). PB kernel only has one parameter
- MB
  - <lat type="Erlang" m="5" r="5/4.17854" />: Length latency (non-infectios) period
  - <incmb type="Constant" c="0.0" />: Length incubation period until MB-disease

- <det type="TimeDistribution" ....>: Time until detection after incubation period
- < kernel type="MBKernel" betalow="0.0" betahigh="1.0" />: Infectivity function (incorrectly called kernel). MBKernel has two parameter and is a straight line between betalow and betahigh. MBstepKernel requires an extra parameter steps, which indicates the number of steps required to reach betahigh. This infectivity function is equivalent to the function in the SIMLEP model.

The name kernel for the infectivity function is chosen badly. This function determines the probability of infection during a contact.

Parameters

<cure_state value="9" />: Determine to which state an individual cures
<selfheal_state value="8" />: Determine to which state an individual self-heals.
States of an individual are:

0. Susceptible
1. Latent
2. Asymptomatic low contagious
3. Symptomatic low contagious
4. Asymptomatic building up contagiousness
5. Symptomatic building up contagiousness
6. Symptomatic highly contagiousness
7. Recovered / Never susceptible state
8. Recovered /Self healed
9. Recovered / Cured

Use of three recovered classes to be able to distinguish between never susceptible, self-healed and cured. Choosing to set self-healing and cure to 7 will result in no relapse.

Susceptibility

The type of leprosy is based on two mechanisms in the simulation program. First the program checks whether the household of the individual can contain susceptibles (see below under “the role of the household”), and secondly by characteristics of the individual. These characteristics are simply the DiseaseHistory attributed to this individual. The DiseaseHistories (see above) are numbered. For example if there are three types of disease, the user should specify DiseaseHistory0, DiseaseHistory1 and DiseaseHistory2.
The parameter iModel than determines through which mechanism these individual characteristics can be distributed over individuals.

The role of the household

Determination of susceptibility always starts with the household. The household needs to be “susceptible”. This is randomly determined when the household is created in the simulation with probability given by parameter “dFactor”. If this parameter is 1 all households are susceptible, and susceptibility of individuals is completely determined by their own leprosy characteristics.

Leprosy characteristics

The leprosy characteristics of an individual are the type of disease this individual will develop after infcetion. The leprosy characterics can be randomly determined, by a selection between the mother or fathers characteristic or by a model of Mendelian inheritance. The outcome is one of the disease types described above for the DiseaseHistory-functions.

Randomly determined

Here a function determines which fraction of the population gets what type of disease.

From parent
Get the exact type of one of the two parents.

Inherit susceptibility
Only inherit susceptibility from your parents. The exact type is determined randomly.

Mendelian inheritance

For Mendelian inheritance the genotypes need to be defined. Therefore first the number of genes need to be given (parameters *genes*) and the number of alleles per genes (parameters *alleles*X where X defines the gene). The number of alleles is the number of possible values one gene can have. Each gene can have the number of distinct values determined by the corresponding alleles parameter. Each individual has a genotype consisting of 2 times the number of genes (one of the father and one of the mother). In the input file the “phenotype”, which is the type of leprosy and susceptibility to leprosy, of each of these possible genotypes needs to be defined.

For example if there is only 1 gene with two alleles (0 and 1) the following genotypes are possible: 00,01,10,11. These can for instance correspond to 00 = not susceptible (<g00 value = “0”/>), 01 and 10 are PB leprosy (<g10 value =”1”/> and <g01 value =”1”/>) and 11 is MB leprosy (<g11 value = “2”/>).

Important! The fraction of each disease type occurring is determined by the gene-frequencies. Due to genetic drift (especially with small populations) the gene-frequencies and thus the fractions of types of disease will change during the simulation. Therefore the initial gene-frequencies should be calibrated to have the required frequencies at the time of interest.

Parameters

<distypes value="3" />	Number of disease types, which are determined by the DiseaseHistory-functions
<dFactor value="0.25" />	For household-model of susceptibility the fraction of households with susceptible inhabitants. Individuals in households that have the “susceptibility factor” can be infected. The susceptibility factor is determined at the moment the household is created.
<iModel value="3" />	Type of model determining the susceptibility

0. random = everybody has the same probability
1. type = only inherit type of parent, susceptibility is random
2. susceptibility = inherit susceptibility, type is random
3. mendelian = inheritance through mendelian genetics

Type and Susceptibility require:

- <dis_mom value="0.5" />:Probability of inheriting the type/susceptibility of the mother

Mendelian requires:

- <genes value="2" />: Number of genes determining susceptibility and type
- <alleles0 value="2" />: Number of alleles of gene 0.
- <gXXYY.. value =”a”/>: Define for each possible genetic combination “XXYY” the phenotype “a”. E.g. for 2 genes with 2 alleles the number of genotypes to define is 2^2 x 2^2 = 16.

Functions

For each gene define the fraction present, take into account that due to genetic drift the fraction of susceptibles can change:

```
<g1 type="QFraction" cum="false" interpolate="false">
  <e min="0" value="0.892" />
  <e min="1" value="0.108" />
</g1>
```

Methods

Sometimes it is required to “mix” mechanisms.

<twosusmechanisms value="true"> With this method on “true” it is possible to have two susceptibility mechanisms in the same population e.g. genetic + household. This input is not required and will then default to “false”.

This requires a method to determine which fraction of those that are not genetically determined have what type:, with in this example 90% of type 0, 8% of type 1 and 2% of type 2

```
<tsmDistribution type="QFraction" cum="false" interpolate="false">
  <e min="0" value="0.90" />
  <e min="1" value="0.08" />
  <e min="2" value="0.02" />
</tsmDistribution>
```

Introduction of infection

Parameters

<imports_per_event value="1" />: New infections per event
<import_events value="50" />: Number of events -
<time_of_firstImport value="xx" />: First import after a burn-in of demography of xx year
<import_method value="2" />:

0. random selection of a person
1. Only susceptibles
2. Only certain type
3. Only certain household size

<import_type value="2" />: Import infections in a type (only method 2)
<import_hhsize value="2" /> Import in household of size (only method 3)

Functions

<TimeOfImport type="Constant" value="2" /> : Time between two import events

Interventions

Methods

<contacttracing value="on" />:	Examine household members of new patient
<followup value="on" />:	Follow the household member for a certain period
<BCGcampaign value="on" />:	Vaccinate children up to a certain age with BCG in a specified time-frame
<prophylactic value="off" />:	Give prophylactics to household members

<contactvaccination value="off" />:	Vaccinate household members
<survey value="off" />:	Do active surveys of (a part of) the population
<blanket value="off" />:	Treat the whole population with anti-leprosy medication

Treatment

Functions

<!--duration of treatment -->

<treatmentduration type=...>:	Duration of treatment. This will have the disease diagnosis as input.

<!--relapse periods-->

<relapse_period0 type=... />:	Time until relapse for each state(0-9) being treated

<!--relapse probabilities-->

<relapse0 type="Constant" c="0.0" />: Probability of relapse for each state (0-9) when treated

Parameters

<tStart value="970" />:	Start treatment
<relapseUpgraded value="0.9" />	Probability of relapse to another type
<relapseType value="X" />	Relapse to type X

Active Detection

Functions

For each state define the probability of active detection for each state (1 to 9). This is a function to allow for active detection increasing for the time since being in a certain state.

<Detection1 type="Constant" c="0.0" />
<Detection2 type="Constant" c="0.0" />
<Detection3 type="Constant" c="0.9" />
<Detection4 etc.

Parameters

<!--contacts-->	
<cStart value="990.0" />:	Start contact tracing
<ccIndividual value="1.0" />:	Probability of complying per person
<ccLevel value="1.0" />:	Probability of complying per household
<ccRank value="1.0" />:	Old stuff do not mind, but keep it here.
<!-- follow up-->	
<fuFreq value="1.0" />	Time between follow ups
<fuEnd value="3.0" />	Time until the end of follow up
<cfIndividual value="1.0" />	Probability of loss to follow up for each cycle.
<!--survey-->	
<sStart value="5000.0" />	Start survey
<sFreq value="2.0" />	Time between surveys
<sEnd value="Infinity" />	End surveying
<scIndividual value="1.0" />	Probability of complying to survey per person

<scLevel value="1.0" />	Probability of complying to survey per household

Methods

<BCGprotection value="on" />:	BCG protects against infection
<BCGshift value="off" />	BCG shifts leprosy-type along spectrum

Functions

<BCGcampaign type="..." />:	Probability of being in BCG campaign given a certain age
<BCGprotection0 type="...” />	BCG protection for each type (0,...)
<BCGcoverage type=...>:	Coverage of BCG at time I

Parameters

<BCGcampaign_start value=../>;	Start BCG campaign
<BCGcampaign_end value=... />	End BCG campaign
<bcgLevel value="0.4" />	Probability of complying per household
<bcgIndividual value="1.0" />	Probability of complying per individual
<bcgFreq value="10.0" />	Time between BCG campaigns
<infBCGshift... value="1.00" />	Shift to another type when vaccinated when in state (0-9)
<BCGxtoy value="0.0" />	Shift between type define for possibility “ x” to “y”
<!-- contact vaccination -->	
<vStart value="5000.0" />	Start vaccination of contacts
<vcLevel value="1.0" />	Probability of complying per household
<vcRank value="0.0" />	Ignore
<vcIndividual value="1.0" />	Probability of complying per individual

<!--CHEMOPROPHYLACTIC TREATMENT-->

Functions

<chemocureX type="Constant" c="0.0" />:	Probability of cure by chemoprophylaxis if treated being in state X (define for 0-9)
<chemodelayX type="Constant" c="0.0" />:	Time of delay of the incubation period by chemoprophylaxis when treated being in state X (define 0-9).
<!-- chemo-->	
<pStart value="5000.0" />:	Start chemoprophylaxis
<pcIndividual value="1.0" />	Probability of individual to complying to chemoprophylaxis
<pcLevel value="1.0" />	Probability of household to complying to chemoprophylaxis
<pcRank value="0" />	Ignore
<!-- blanket -->	
<bStart value="5000.0" />	Start blanket treatment
<bFreq value="50.0" />	Time between blanket treatments
<bEnd value="Infinity" />	End of blanket treatment

<bcIndividual value="1.0" />	Probability of individual to comply to blanket treatment
<bcLevel value="1.0" />	Probability of household to comply to blanket treatment

TABLES' for DEMOGRAPHY

Functions

<!-- weight function for mothers -->

<w_birth type=...>:	Weight function to place new born in household of female of certain age.
<MarriedMales type=.>	Fraction males married per age class
<MarriedFemales type=.>	Fraction females married per age class
<malesurvival type=...>	Survival curve for males
<femalesurvival type=...>	Survival curve for females

NEIGHBOURHOOD

The SIM*CO*LEP program was at first set up to take into account neighbours etc. This is never quantified and therefore not well tested.

Parameters

<level_depth value="0" />:	Number of groupings (0 = only households)
<virtual_sizex value="Infinity" />:	Maximum size of a "level", i.e. the number of other levels that can be contain by a level of category *x*.

INITIAL DEMOGRAPHIC DISTRIBUTIONS

The initial distributions of age of males, females and household sizes are required. These distributions could be chosen randomly after which the simulation is run for an extended times until a quasi steady state has been reached. To reduce computation time these distributions can be given such that these are near the quasi steady state. The value can be obtained by running the model for a long period (e.g. 5000 years) with the demographic parameters as these are in the beginning of a simulation (e.g. no population growth, no changes in survival etc.). Using the value of such a run (or the average of a number of runs) can be used to set the initial distributions.

Functions

<initagedist_male type=...>:	Initial age distribution for males-->
<initagedist_female type=...>:	Initial age distribution for females-->
<initlevelsize type=...>:	Initial distribution of household sizes

Seeds

<pop>935</pop>	Seed for random number generator of population module
<dis>80523</dis>	Seed for random number generator of disease module
<intro>603037</intro>	Seed for random number generator of introduction of infections

Settings

Methods

<GUI value="off" />	Show progress bar (turn off because it slows down simulation
<dialog value="off" />	Give a dialog to report errors. For multiple runs turn off, because otherwise it will not continue.
<timemeasurer value="off" />	Measure real time per run, slows down simulation
<clockseed value="off" />	Use clock value to generate seeds instead of seeds
<overwriteAll value="on" />	Overwrite all existing outputfiles without warning
<one_file value="on" />	Record all to one file. If off all outputs will be written to a separate files
<safemode value="off" />	For debugging.
<savehist value="off" />	Remember individual histories, turn off in non-debugging mode as it will slow down simulation
<saveevents value="off" />	Remember events, turn off in non-debugging mode as it will slow down simulation

Parameters

<step value="4.5/12" />	Step size discrete updated values of fraction married, and births for population growth.
<ageclassmax value="105" />	Initial maximum age
<ageclasswidth value="5.0" />	Class width of population register for output

These settings give an optimal performance of the simulation using the EventManager. The eventmanager handles the events of the simulation. The EventManager is equal to the EventManager of STDSIM (version 2005).

<!--EventManager settings-->
<bucketSize value="16384" />
<bucketWidth value="10000000001" />
