# Supplementary material for: Global elimination of leprosy by 2020: are we
Source: Parasit Vectors. 2015 Oct 22;8:548. doi: 10.1186/s13071-015-1143-4 (PMC4618543; doi:10.1186/s13071-015-1143-4)
Supplement: Additional file 3: — SIMCOLEP model with input files. Zip archive contains: Instruction file, SIMCOLEP model (version 1.4.12), Input files for India, Brazil and Indonesia, Source code, R-script for output. The contents of this zip archive are licensed under the Creative Commons Attribution-NonCommercial-NoDerivatives 4.0 International License. To view a copy of this license, visit http://creativecommons.org/licenses/by-nc-nd/4.0/ or send a letter to Creative Commons, PO Box 1866, Mountain View, CA 94042, USA. By opening Additional file 3, you agree to the aforementioned license. You are free to use and share (copy and redistribute the material in any medium or format) the material contained within Additional File 3 under the following terms: Attribution — You must give appropriate credit, provide a link to the license, and indicate if changes were made. You may do so in any reasonable manner, but not in any way that suggests the licensor endorses you or your use. NonCommercial — You may not use the material for commercial purposes. NoDerivatives — If you remix, transform, or build upon the material, you may not distribute the modified material. [file 13071_2015_1143_MOESM3_ESM.zip › Instruction file.pdf]

Additional file 3: Instructions for using SIMCOLEP (Leprosy model)

David J. Blok[1,]*, Sake J. de Vlas[1], Jan Hendrik Richardus[1]

[1]Department of Public Health, Erasmus MC, University Medical Center Rotterdam, The Netherlands

*Corresponding author: d.j.blok.1@erasmusmc.nl

Description

SIMCOLEP is an individual-based model (or microsimulation) developed by Erasmus MC. The model was written in JAVA. It simulates the spread of *M. leprae* in a population of individuals that are structured in households.

Running SIMCOLEP

Requirements: latest Java runtime installed on your computer.

Instructions:

1. Place following files in one folder:
    - Batch-file: *RUNALL.bat*
    - JAR-file: *SIMCOLEP_ 1_4_12.jar*
    - Input files (XML): *lep_india.xml, lep_brazil.xml, lep_indonesia.xml*
2. To run the model double click the batch-file

Source code

The folder 'Source code' contains the source code of SIMCOLEP version 1.4.12.

Input

The folder 'Input' contains the input files for India, Brazil and Indonesia. The SIM*CO*LEP input file is an xml-file that can be edited with any text editor or alternatively, with an XML editor (such as Oxygen XML Editor). A detailed description of the input file can be found in Additional file 2: SIMCOLEP manual.

Output

The folder 'Output' contains an R script to analyze the output of the model together with an example output file. Output can to some extend be specified in the input file (see Additional file 2: SIMCOLEP manual). SIMCOLEP generates two output files: CSV-file (individual results) and txt-file (aggregated results). The names of the output files can be specified in the input file.

For this study we only used the aggregated results. The txt-file includes many tables, of which the following are of interest: "DgaMale", "DgaFemale", "Dhah", "Lia i_diag1", and "Lia i_diag3". These tables provide information about the number of males and females, number of people per household size, the number of people diagnosed with PB, and the number of people diagnosed with MB. The provided R script (*simcolep_output.R*) will extract the correct tables and generate the results as provided in the manuscript.
