# Supplementary material for: Global elimination of leprosy by 2020: are we
Source: Parasit Vectors. 2015 Oct 22;8:548. doi: 10.1186/s13071-015-1143-4 (PMC4618543; doi:10.1186/s13071-015-1143-4)
Supplement: Additional file 3: — SIMCOLEP model with input files. Zip archive contains: Instruction file, SIMCOLEP model (version 1.4.12), Input files for India, Brazil and Indonesia, Source code, R-script for output. The contents of this zip archive are licensed under the Creative Commons Attribution-NonCommercial-NoDerivatives 4.0 International License. To view a copy of this license, visit http://creativecommons.org/licenses/by-nc-nd/4.0/ or send a letter to Creative Commons, PO Box 1866, Mountain View, CA 94042, USA. By opening Additional file 3, you agree to the aforementioned license. You are free to use and share (copy and redistribute the material in any medium or format) the material contained within Additional File 3 under the following terms: Attribution — You must give appropriate credit, provide a link to the license, and indicate if changes were made. You may do so in any reasonable manner, but not in any way that suggests the licensor endorses you or your use. NonCommercial — You may not use the material for commercial purposes. NoDerivatives — If you remix, transform, or build upon the material, you may not distribute the modified material. [file 13071_2015_1143_MOESM3_ESM.zip › Source code/simcolep/package.html]

SIMCOLEP: simulation of contacts of leprosy patients
